# Effect of *Cistanche Tubulosa* Extracts on Male Reproductive Function in Streptozotocin–Nicotinamide-Induced Diabetic Rats

**DOI:** 10.3390/nu10101562

**Published:** 2018-10-22

**Authors:** Zwe-Ling Kong, Athira Johnson, Fan-Chi Ko, Jia-Ling He, Shu-Chun Cheng

**Affiliations:** Department of Food Science, National Taiwan Ocean University, Pei-Ning Road, Keelung 20224, Taiwan; athirajohnson07@gmail.com (A.J.); makeadrea@hotmail.com (F.-C.K.); k46255@gmail.com (J.-L.H.); riceroll826@gmail.com (S.-C.C.)

**Keywords:** diabetes, infertility, *Cistanche tubulosa* extract (CTE), echinacoside (ECH), anti-inflammatory activity, antioxidant activity, steroidogenesis effects

## Abstract

Diabetes is a chronic disorder characterized by hyperglycemia due to decreased levels of insulin or the inefficiency of the tissue to use it effectively. Infertility is known as a major outcome of diabetes and affects the male reproductive system by causing sperm impairment and gonadal dysfunction. *Cistanche tubulosa* is a parasitic plant which has the capacity to improve memory, immunity, and sexual ability, reduce impotence, and minimize constipation. This study was focused on the investigation of the anti-inflammatory and protective effects of echinacoside (ECH) in *Cistanche tubulosa* extract (CTE) on the male reproductive system of the diabetic rats. The antioxidant, anti-inflammatory, and protective effects of CTE were evaluated by both in vitro and in vivo methods. The in vitro results show that the ECH inhibited reactive oxygen species (ROS) production and improved StAR, CYP11A1, CYP17A1, and HSD17β3 protein expression. The in vivo analysis was carried out with three doses of echinacoside (ECH) (80, 160, and 320 mg/kg) in CTE. In total, 0.571 mg/kg of rosiglitazone (RSG) was administered as a positive control. Diabetes was induced by streptozotocin (STZ) (65 mg/kg) and nicotinamide (230 mg/kg) in combination with a high-fat diet (45%). The in vivo studies confirmed that the ECH improved blood sugar levels, insulin resistance, leptin resistance, and lipid peroxidation. It can restore kisspeptin 1 (KiSS1), G protein-coupled receptor GPR 54, suppressor of cytokine signaling 3 (SOCS-3), and sirtuin 1 (SIRT1) messenger ribonucleic acid (mRNA) expression in the hypothalamus and recover sex hormone level. Thus, this study confirmed the antioxidant, anti-inflammatory, and steroidogenesis effects of CTE.

## 1. Introduction

Diabetes mellitus (DM) is a condition in which there are increased glucose levels in the blood due to the inefficiency of the pancreas in producing enough insulin, or the inability of the body to use it effectively. According to the WHO, the number of people with diabetes rose from 108 million in 1980 to 422 million in 2014 [1]. There are four main categories: pre-diabetes (a stage prior to diabetes), type 1 (where the pancreas fails to produce insulin), type 2 (the body fails to use insulin), and gestational diabetes (which occurs during pregnancy). Oxidative stress occurs due to the imbalance between the production of reactive oxygen species (ROS) and antioxidants [2] finally leads to the diabetic condition. The complications of diabetes include failure of the kidney, nerve damage, blindness, stroke, heart attack, fetal death, and infertility. [1]. Studies show that sperm nuclei, deoxyribonucleic acid (DNA), and mitochondria were significantly damaged in male diabetic patients [3]. Oxidative stress during the transportation of sperm will alter the process of the male reproductive system [4,5].

The process of spermatogenesis is controlled by hypothalamus–pituitary–gonadal (HPG) axis. The gonadotropin-releasing hormone (GnRH) produced by the hypothalamus stimulates the production of luteinizing hormone (LH) and follicle-stimulating hormone (FSH). Leydig cells are found adjacent to the seminiferous tubule and produce testosterone under the control of LH. FSH triggers the production of antigen-binding protein (ABP) that helps to pass the male hormone. Thus, infertility and other problems mainly arise due to the disturbances that occur in the HPG axis. Administration of the combination of streptozotocin–nicotinamide induces diabetes in experimental rats. Streptozotocin is a chemical compound toxic to insulin-producing β cells in the pancreas and nicotinamide is a water-soluble vitamin that protects the β cells from complete damage [6].

*Cistanche tubulosa* is a desert plant species also known as “Rou Cong-Rong” [7]. It is a non-chlorophyllic parasitic plant that grows mainly on the root of the *Calotropis procera* tree and is widely distributed in the arid land of Gansu, Qinghai, Xinjiang, Mongolia, Iran, and India. The common name of *Cistanche tubulosa* is “Desert Hyacinth”. It is widely accepted in Chinese traditional medicine and has been given the name “Ginseng of the Desert”. It is widely used in the medicinal field to treat morbid leucorrhea, profuse metrorrhagia, chronic renal diseases, constipation, impotence, and infertility. Chemical constituents of *Cistanche tubulosa* consist of non-volatile phenylethanoid glycosides (PHGs), iridoids, lignans, volatile oils, alditols, oligosaccharides, and polysaccharides [8]. Studies show that echinacoside from this herb protects the damaged fibroblast by regulating ROS levels [9]. This compound produces antioxidant, vasorelaxation, and anti-inflammatory activities together with neuroprotection and osteoporosis prevention [10,11].

This study was aimed to investigate the effect of *Cistanche tubulosa* extract containing ECH on the reproductive dysfunction of the streptozotocin–nicotinamide-induced diabetic rat.

## 2. Materials and Methods

### 2.1. Materials

ECH and CTE were purchased from Sinphar Pharmaceutical Co., Ltd (Yilan, Taiwan). Advanced glycation endproducts (AGEs) were purchased from Biovision (San Francisco, CA, USA). LC-540 and TM3 cell lines were purchased from the Food Industry Research and Development Institute (Hsinchu, Taiwan). Five-week-old Sprague-Dawley (SD) male rats were purchased from the National Laboratory Animal Center (Taipei, Taiwan). Feed Lab Diet^®^ was purchased from PMI Nutrition International, Inc. (Taipei, Taiwan). 2,2-diphenyl-1-picrylhydrazyl (DPPH) was obtained from Sigma (St. Louis, MO, USA). Methanol and 4-(2-hydroxyethyl)-1-piperazineethanesulfonic acid (HEPES) were also purchased from Sigma. Dulbecco’s modified Eagle medium/F12 and trypsin-EDTA were obtained from GIBCO (New York, NY, USA). 3-(4, 5-dimethylthiazol-2-yl)-2, 5-diphenyltetrazolium bromide (MTT), dichloro-dihydro-fluorescein diacetate (DCFH-DA), dimethyl sulfoxide (DMSO), and nitroblue tetrazolium (NBT) were purchased from Sigma. Glucose enzymatic kits were obtained from Kyokutoseiyaku, Tokyo, Japan. Insulin ELISA kits were purchased from Mercodia AB Inc., Sylveniusgatan 8A (Uppsala, Sweden). T-PER Tissue Protein Extraction Reagent was acquired from Thermo Scientific (Chicago, IL, USA). Anti-Human/Mouse/Rat nuclear factor-kappa B NF-κB polyclonal antibodies were purchased from Santa Cruz Biotechnology, Santa Cruz, CA, USA. Anti-Mouse/Rat receptor for advanced glycation endproducts (RAGE) polyclonal antibodies, anti-Human/Mouse/Rat StAR polyclonal antibodies, and anti-Human/Mouse/Rat Cytochrome P450 17A1 monoclonal antibodies were purchased from Gene Tex (Irvine, CA, USA). Anti-Human/Mouse/Rat CYP11A1 polyclonal antibodies were obtained from Cell Signaling Technology (Beverly, PA, USA). Easy-Blue reagent was purchased from Invitrogen, Thermo Fisher Scientific (Carlsbad, CA, USA). Agarose and DNA marker 100 bp were obtained from Promega, Corporation (Madison, WI, USA). RNeasy Lipid Tissue Mini Kit was purchased from QIAGEN, Hilden, Germany.

### 2.2. Methods

#### 2.2.1. In Vitro Analysis

LC-540 and TM3 Cell Culture: The LC-540 cell lines were cultured at 37 °C in Earle’s Balanced Salt Solution (EBSS) medium supplemented with sodium bicarbonate (1.5 g/L), l-glutamine (2 mM), non-essential amino acids (0.1 mM), sodium pyruvate (1.0 mM), and fetal bovine serum (FBS) (10%) in a 5% CO_2_ incubator (CO_2_ incubator, Napco 5410, Taiwan). The TM3 cell line was cultured in Dulbecco’s modified Eagle medium (DMEM) supplemented with glucose (4.5 g/L), sodium pyruvate (0.5 mM), l-glutamine (2.5 mM), sodium bicarbonate (1.2 g/L), 4-(2-hydroxyethyl)-1-piperazineethanesulfonic acid (HEPES, 15 mM), and fetal calf serum (FCS, 10%) or horse serum (5%) at 37 °C in a 5% CO_2_ incubator.

Determination of the Cell Viability of Echinacoside (ECH): 3-(4, 5-dimethylthiazol-2-yl)-2,5-diphenyltetrazolium bromide (MTT) reagent was used for cell viability assay. The cell concentration was adjusted to 2 × 10^5^ cells / mL in a 96-well plate. Then, 20 μL of ECH (diluted in 2% of medium) was added and incubated for 24 h at 37 °C in a 5% CO_2_ incubator. Later, 100 μL of MTT reagent was added and incubated for 4 h at 37 °C under dark conditions. The absorbance was measured at 570 nm (ELISA reader, Dynatech MR5000, Kloten, Switzerland).

Relative cell viability (%) = [(A_sample at 570 nm_ − A_blank at 570 nm_)]/[(A_control at 570_ − A_blank at 570_)] × 100.

Nitroblue Tetrazolium (NBT) Reduction Assay and 2,2-diphenyl-1-picrylhydrazyl (DPPH) Assay: The number of cells was adjusted to 1× 10^6^ cells/mL. Then, 50 mg/mL of ECH, resveratrol (RES), and advanced glycation end-products (AGEs) (control) were added and co-cultured for 18 h at 37 °C. Then, 0.3 mL of NBT solution (0.1 mg/mL NBT, 5% FBS, and 3% dimethyl sulfoxide (DMSO) dissolved in 10 mL DMEM) was added and incubated at 37 °C in 5% CO_2_ for 1 h. After centrifugation (at 800× *g* for 3 min) the supernatant was removed and 200 μL DMSO was added and shaken for 5 min in an ultrasonic oscillator (Delta Ultrasonic Cleaner D 200, Keelung, Taiwan). The absorbance was measured at 630 nm. For DPPH radical assay, Trolox (in 95% alcohol) was taken as the standard. Then, 75 μL of 0.5 mM DPPH solution was mixed with 25 μL of samples and allowed to react at room temperature in a dark place for 30 min. The mixture was shaken and the absorbance was measured at 517 nm.

Reactive Oxygen Species (ROS) Content Analysis: The cell number was adjusted to 2 × 10^5^ cells/mL in a 12-well plate containing 2% FBS. Then, 50 μL/mL of ECH, RES, and AGEs were added to the plate and incubated at 37 °C for 24 h. After 24 h, 2′,7′-dichlorofluorescein-diacetate (DCFH-DA) was added and incubated at 37 °C for 30 min. After centrifugation (400× *g*, 5 min), the supernatant was removed and the cells washed twice with phosphate-buffered saline (PBS). After, cells were suspended in 1 mL of PBS and the levels of ROS were determined by using a flow cytometer (Flow Cytometer, Becton Dickinson, CA, USA).

Identification and Quantitative Analysis of Protein: Proteins were extracted by using protein extraction reagent (T-PER). The cell density was adjusted to 2 × 10^5^ cells/mL in a 12-well plate and 50 μL/mL of ECH, RES, and a receptor for the advanced glycation endproduct (RAGE) antagonist, and AGEs were added and incubated at 37 °C in a 5% CO_2_ incubator for 24 h. After, 400 μL of T-PER were added, scraped off the cells, and centrifuged at 125,000× *g* for 20 min (4 °C). The supernatant was collected and stored at −80 °C (−80 °C freezer, Nuaire, Plymouth, MN, USA) for further analysis. Bicinchoninic acid (BCA) kit was used for protein quantification. Then, 10 μL of standard cell solution and 200 μL of BCA reagent were added to 8-well plates and incubated at 37 °C in a 5% CO_2_ incubator for 30 min. The absorbance was measured at 562 nm. Protein identification analysis was performed by sodium dodecyl sulfate (SDS)-polyacrylamide gel (SDS-PAGE) electrophoresis. Samples were prepared by adding protein lysate to protein loading buffer and western blot analysis was performed to detect the specific proteins.

#### 2.2.2. In Vivo Analysis

Animal Model Design: Sixty 4-week-old male Sprague–Dawley (SD) rats were purchased from the National Laboratory Animal Center (Taipei, Taiwan). Each rat was housed individually in disinfectant stainless steel cages under controlled temperature (23 ± 1 °C) and humidity (40–60%) with 12 h light/12 h dark cycle. Food and water were provided ad libitum. Each rat was domesticated in the first week and given laboratory rodent diet 5001 as the main diet. All procedures followed the standard of Institutional Animal Care and Use Committee (IACUC Approval No. 101026) of the National Taiwan Ocean University, Taiwan. After domestication, rats were divided into two groups: the control group (Con) that was fed with lab diet 5001, and the diabetic group that was fed with a high-fat diet (HFD, 40%) for the entire experiment. After being fed with the HFD for 4 weeks, the diabetic group rats were injected with streptozotocin (65 mg/kg) (STZ) to induce diabetes mellitus (DM). Within 15 min of injecting STZ, nicotinamide (230 mg/Kg Body Weight) was injected. A week after injection, an oral glucose tolerance test (OGTT) was conducted to determine the successful induction of diabetic mellitus (DM). After that, the animals were divided into six groups: the control (fed with lab diet 5001); DM group (DM + 45% HFD); DMR group (DM + rosiglitazone: 0.571 mg/kg BW) + 45% HFD; DME1 group (DM + CTE: 80 mg/kg BW) + 45% HFD; DME2 group (DM + CTE: 160 mg/kg BW) + 45% HFD; and DME4 group (DM + CTE: 320 mg/kg BW) + 45% HFD. Rosiglitazone (RSG) was taken as the positive control. Three doses of CTE (80, 160, and 320 mg/kg) were used according to the recommendation of the Taiwan Food and Drug Administration (TFDA) health food functional evaluation guidelines. Treatments were given until the end of the experiment. All experimental rats were sacrificed after 6 weeks. The blood was collected from the rats was centrifuged at 3000 rpm for 15 min at 4 °C. The supernatant was examined for the following analysis:

Total Glucose, Triglyceride, and Cholesterol Determination: The glucose determination was carried out by glucose enzymatic kits. Then, 20 μL of blood plasma samples were added to the reagents and kept at 37 °C for 5 min. The total triglycerides and total cholesterol concentration were determined by using triglyceride and cholesterol enzymatic kits. Then, 10 μL of blood plasma were added to the reagents and the experiment was conducted according to the manufacturer’s instruction. The absorbance was measured at 510 nm.

Plasma total glucose/triglyceride/cholesterol (mg/dL) = (A_sample_ − A_Blank_)/(A_standard_ − A_Blank_) × 200.

A_sample_: Absorbance of blood samples, A_Blank_: Absorbance value of kits without a sample, A_standard_: Absorbance value of the standard reagent, 200: standard reagents at a concentration of 200 mg/dL.

Insulin, Leptin, and Homeostatic Model Assessment–Insulin Resistance (HOMA-IR) Determination: The insulin level was determined by insulin ELISA kits. Here, 25 μL of blood plasma were added to the reagents and the absorbance was measured at 450 nm. Total leptin content was determined by using leptin enzyme immunometric assay kit. Then, 100 μL of plasma were analyzed and the absorbance was measured at 450 nm using an ELISA reader. The whole experiment was conducted according to the manufacturer’s instruction. The HOMA-IR value was determined from the homeostasis model assessment equation = Fasting plasma insulin concentration (mU/mL) × fasting plasma glucose concentrations (mmol/L)/22.5.

Plasma Lipid Peroxidation: Here, 0.5 mL of blood plasma were mixed with 1 mL of reagent (15%, w/v trichloroacetic acid in 0.25 N hydrochloric acid (HCl) and 0.375%, *w*/*v* thiobarbituric acid in 0.25 N HCl) and placed in a water bath (Water Bath, BUCHI 461, Zürich, Switzerland) at 100 °C for 15 min. After cooling, 1 mL of n-butanol was added, shaken vigorously, and centrifuged at 1500× g for 10 min. The supernatant was collected and the absorbance was measured at 532 nm [12].

Malondialdehyde (MDA) concentration (nM/mL) = [(A_sample at 532 nm_ − A_blank at 532 nm_)/(A_standard at 532 nm_ − A_blank at 532 nm_)] × 5.

Determination of Plasma Testosterone and Luteinizing Hormone (LH) Levels: The testosterone ELISA kit was used to measure the level of testosterone in the blood plasma. RIA kit was used to determine the LH concentration. Then, 50 μL of plasma were added and the calculations were based on the concentration of the hormone LH-RP-3 (standard). The further steps were conducted according to the manufacturer’s instructions.

Determination of Tumor Necrosis Factor (TNF)-α and Interleukin-6 (IL-6) Concentration: The captured antibody was diluted 250 times in coating buffer, added to a 96-well plate (100 μL/well), and kept at 4 °C for overnight. The supernatant was aspirated and washed five times with washing buffer (1 time PBS, 0.05% Tween 20). After incubation (1 h) with 200 μg of assay diluent, the cells were washed 5 times with washing buffer and 100 μL of TNF-α standard solution or test samples were added to each well and incubated for 2 h. After washing, 100 μL of IL-6 detected antibody were added and incubated for 1 h at room temperature. The supernatant was aspirated and washed 5 times. Then, 480 μL of enzyme avidin-horseradish peroxidase (HRP) were added and incubated for 30 min at room temperature. The supernatant was aspirated and washed five times with washing buffer. Then, 100 μL of substrate solution were added and incubated for 15 min at room temperature. Later, 50 μL of stop solution (1M phosphoric acid) were added to each well and the absorbance measured at 450 nm.

Analysis of Sperm and Testis Parameters

Sperm Sample Collection: The sperm sample collection was performed according to the method previously reported in [13]. The sperm were collected from the supernatant and used for further analysis.

The Number of Sperm, Sperm Motility, and Abnormal Sperm: The number of sperm were calculated using the hemocytometer and trypan blue solution. Then, 100 μL of sperm liquid were mixed with trypan blue solution and the number of sperm, motility, and abnormal sperm were calculated using hemocytometer and microscope [14].

Nitro blue Tetrazolium NBT Reduction and Lipid Peroxidation of Sperm and Testis: The lipid peroxidation and NBT reduction were analyzed according to the same procedure of plasma analysis.

Determination of Superoxide Dismutase (SOD) and Catalase Activity: Superoxide dismutase (SOD) activity was analyzed by using the RANSEL kit. Here, 0.05 mL of testicular homogenates were added to 1.7 mL of reaction solution (0.05 mM xanthine, 0.025 mM 2-(4-iodophenyl)-3-(4-nitrophenol)-5-phenyltetrazolium chloride (INT)). After mixing, 0.25 mL xanthine oxidase (80 U/L) was added and reacted at room temperature for 30 s at room temperature. The absorbance was measured at 505 nm. The protein concentration of the testicular homogenate was determined by Bio-Rad DC protein assay kit and its specific activity (U/mg protein) was calculated. The catalase (CAT) activity was determined according to a previously reported method [15].

H&E (Hematoxylin and Eosin) Staining

The testis was soaked in 10% of formalin for 24 h and stored at 4 °C. Sliced the tissues into the micro size and attached to the slide. After fixation (95% methanol + 5% acetic acid), the slides were immersed in hematoxylin for 3 min, washed with running water for 5 min, and then soaked them with 50%, 70%, and 90% alcohol for one minute. After that, the slides were stained with eosin for 10 s and soaked in 100% alcohol for one minute until it faded. Finally, the slide was soaked in xylene for one minute. Later, the slide was air-dried and sealed. The stained tube was placed in an inverted phase contrast microscope (Inverted Phase Difference Microscope, Olympus CK-2, Tokyo, Japan) to observe the morphology.

Analysis of the Hypothalamus

The mouse KISS1, G-protein coupled receptor (*GPR) 54, suppressor of cytokine signaling 3 (SOCS-3) and sirtuin 1(SIRT1)* gene sequences were identified from the NCBI (National Center for Biotechnology Information) gene database and a specific primer was designed using Primer 5.0 PREMIER Biosoft, Palo Alto, CA, USA.). The primers used in the experiment are listed in the Table 1.

Extraction of Ribonucleic acid (RNA) from the Hypothalamus. The extraction of RNA from the hypothalamus of the brain was carried out by using the RNeasy Lipid Tissue Mini Kit. The obtained mRNAs were quantitatively measured by reverse transcription (RT) and polymerase chain reaction (PCR). The compliment DNA obtained from RT analysis was stored at −20 °C for later use. The extracted DNA was subjected to polymerase chain reaction (PCR) analysis using Taq polymerase. Then, 5~10 μL of the PCR product were taken and analyzed by electrophoresis on a 1.5% agar colloid. The image was taken in a UVP BioDoc-It imaging system. Reverse-transcribed complimentary deoxyribonucleic acid (cDNA)was taken and PCR was performed using the IQ SYBR Green Supermix Kit. Later, it was analyzed with iQTM 5 Optical System Software. Proteins were extracted by using protein extraction reagent (T-PER). Then, 20 mg of testicular homogenate were added to 400 μL of T-PER and centrifuged (High Speed Centrifuge, Hettich CR-12, Tuttlingen, Germany) at 4 °C (125,000× *g*) for 20 min. The following method was similar to” identification and quantification analysis of protein” in in vitro analysis.

### 2.3. Statistical Analysis

The experimental results were expressed as the mean ± standard deviation (Mean ± SD) and the data were analyzed using Statistical Product & Service Solutions (SPSS) 11.0 software, IBM, Armonk, New York, NY, USA. The differences between the groups were analyzed by one-way analysis of variance (ANOVA). Multiple comparisons of different groups were analyzed by Duncan’s test at the value of *p* < 0.05 as the significant level.

## 3. Results

### 3.1. In Vitro Analysis

#### 3.1.1. Comparison of Antioxidant Activities of ECH, CTE, and RES

**Figure 1:**
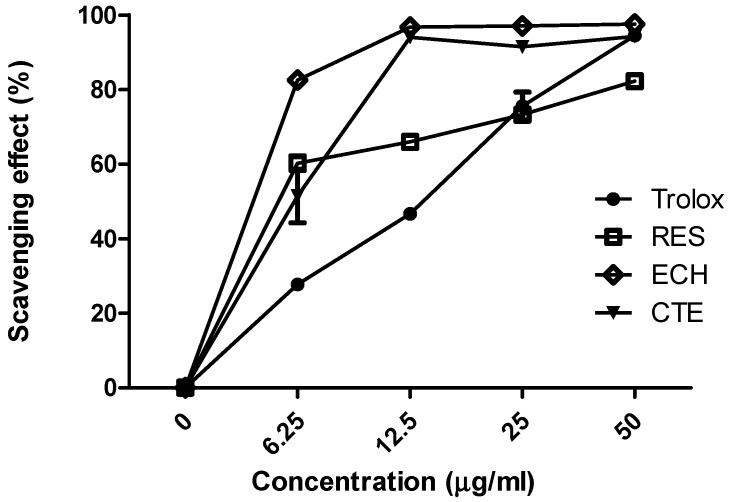
2,2-diphenyl-1-picrylhydrazyl (DPPH) radical scavenging effect of Trolox (●), RES (□), ECH (◇), and CTE (▼). Results were shown as mean ± SD (*n* = 3). (ECH: echinacoside; RES: resveratrol; CTE: *Cistanche tubulosa* extract).

Long-term high oxidative stress and chronic inflammation are the main causes of diabetic complications [16]. CTEs contain various phenylethanoid glycosides such as echinacoside (ECH) and acteoside, with the potential for antioxidant activity [17]. DPPH radical scavenging assay was conducted for evaluating the antioxidant activity of ECH. Trolox and resveratrol (3,5,4’-trihydroxystilbene, RES) were taken as the standard and positive control respectively. As shown in Figure 1, ECH showed better radical scavenging activity and the activity was significantly higher than that of the positive control (RES).

#### 3.1.2. Cell Viability of ECH on LC-540 and TM3 Leydig Cells

**Figure 2:**
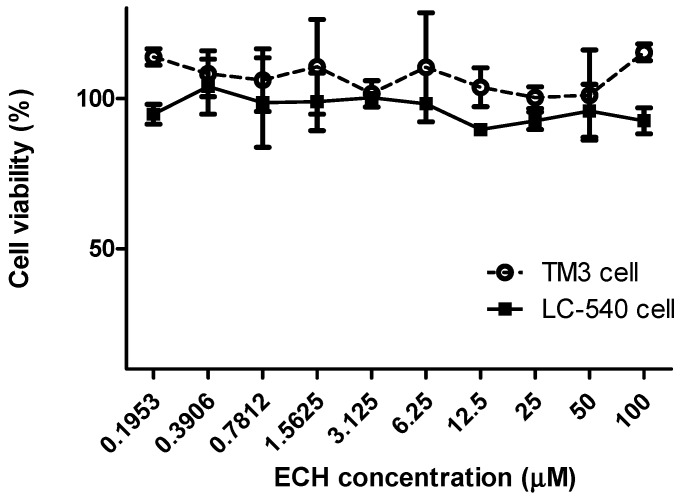
The cell viability of different concentration of ECH in LC-540 and TM3 Leydig cells. Cell viability was examined by 3-(4,5-dimethylthiazol-2-yl)-2,5-diphenyltetrazolium bromide (MTT) assay. The cell number was adjusted to 2 × 10^5^ cells/mL, and treatment with ECH was given for 24 h. Results were shown as mean ± SD (*n* = 3).

MTT assay was performed for evaluating the cell viability of different concentration of ECH. LC-540 Leydig cells and TM3 Leydig cells showed more than 80% of viability after being treated with ECH (Figure 2). As compared to the other concentrations, a 100-μM concentration (dilutions of 100 μM in 2% FBS) of ECH showed better cell viability in TM3 Leydig cells. The result indicated that the increased concentration of ECH does not contribute any significant toxicity to the cells.

#### 3.1.3. Effect of ECH on AGE-Induced Superoxide Production by NBT Assay in LC-540 and TM3 Leydig Cells

**Figure 3:**
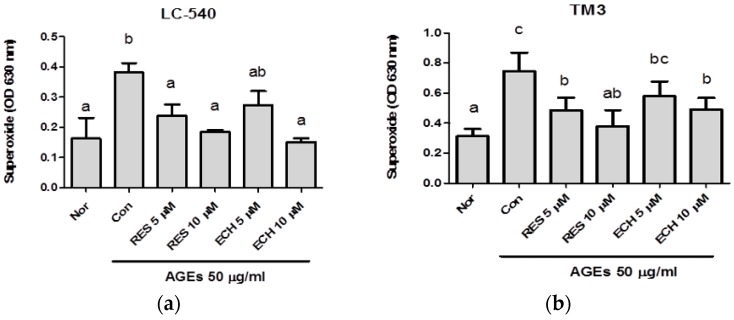
Effect of ECH and resveratrol on advanced glycation endproduct (AGE)-induced superoxide production by nitroblue tetrazolium (NBT) assay in LC-540 (**a**) and TM3 Leydig cells (**b**). The cell number was adjusted to 2 × 10^5^ cells/mL. The normal group (Nor) was treated with medium, and the control group (Con) was treated with AGEs (50 μg/mL). Doses of 5 and 10 μM of RES and ECH were tested. Results were shown as mean ± SD (*n* = 3). The significant differences (*p* < 0.05) were marked with different letters (a–c).

AGEs cause oxidative damage in the body due to the oxidation of glucose and the formation of free radicals such as O_2_-, hydroxyl radicals, and carbonyl groups [18]. After being stimulated with 50 μg/mL of AGEs, the LC-540 (Figure 3a) and TM3 (Figure 3b) cells were treated with ECH and RES (5 μM and 10 μM each). The results showed that the production of superoxide anion was increased in the control group (stimulated AGEs), but the production of superoxide anion was decreased after treatment with ECH and RES. From Figure 3, it was understood that the ECH and RES reduce the level of superoxide production and protects the cells from oxidative damage. In the case of LC-540 cells, 10 μM ECH showed almost similar activity with the normal group. The superoxide production in both cells was decreased with an increase in the concentration of both ECH and RES.

#### 3.1.4. Effect of ECH on H_2_O_2_ Production in AGE-Stimulated LC-540 Leydig Cells

**Figure 4:**
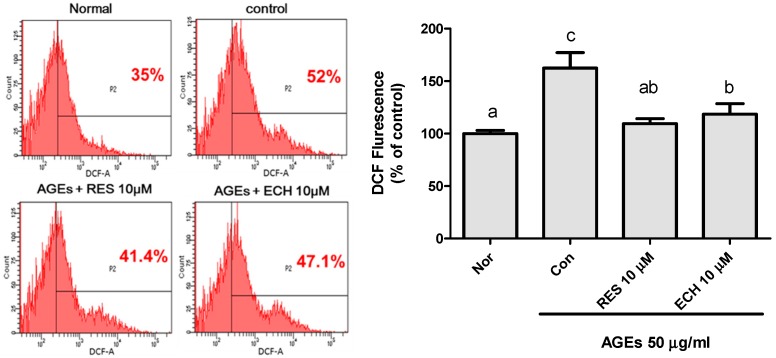
Effect of ECH and resveratrol on H_2_O_2_ production in AGE-stimulated LC-540 Leydig cells by flow cytometer dichloro-dihydro-fluorescein diacetate (DCFH-DA. The cell number was adjusted to 2 × 10^5^ cells/mL. Here, 10 μM of RES and ECH were added for analysis. The normal group (Nor) was treated with medium, and the control group was treated with 50 μg/mL of AGEs. The significant differences (*p* < 0.05) were marked with different letters (a–c).

DCFH-DA assay was executed for detecting the presence of oxidative species. After entering into the cells, the fluorescent dye DCFH-DA is oxidized by intracellular H_2_O_2_ and forms dichlorofluorescein (DCF). As shown in Figure 4, there was a significant increase in intracellular H_2_O_2_ production in the AGE-stimulated group (control) whereas the addition of 10 μM ECH and RES significantly reduced the H_2_O_2_ production in cells. Administration of 10 μM ECH resulted in only about 47.1% of H_2_O_2_ production, significantly lower than in the control group.

#### 3.1.5. Effect of ECH on RAGE and NF-κB Protein Expression Levels in AGE-Stimulated LC-540 Leydig Cells

**Figure 5:**
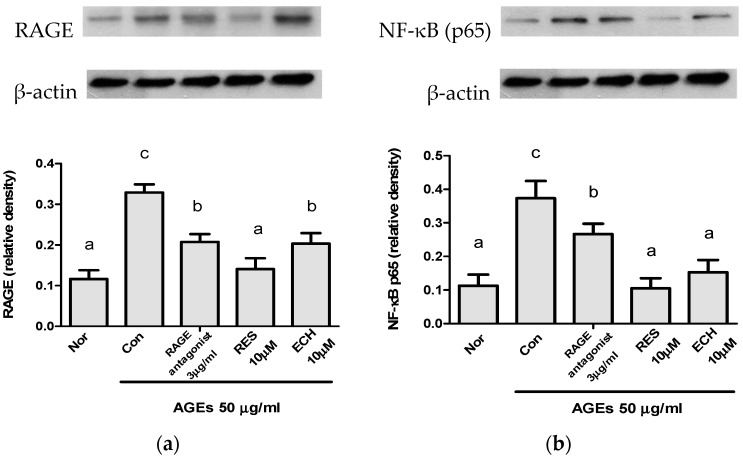
Effect of ECH on (**a**) RAGE and (**b**) NF-κB protein levels in AGEs- stimulated LC-540 Leydig cell. The cell number was adjusted to 2 × 10^5^ cells/mL and treated with RES and ECH (10 μM) for 24 h. β-actin was taken as the internal control. Normal group (Nor) was treated with medium and control group (Con) were treated with the AGEs (50 μg/mL). Results were shown by mean ± SD (*n* = 3). Significantly differences (*p* < 0.05) were represented by letters (a–c) analyzed by Duncan’s multiple range test.

Western blot analysis was performed to confirm the presence of RAGE in LC-540 Leydig cells. The effect of ECH on RAGE (Figure 5a) and NF-κB (Figure 5b) protein expression levels in AGEs-stimulated Leydig cells were shown in Figure 5. The results showed that, 50 μg/mL concentration of AGEs induced higher RAGE and NF-κB expression in LC-540 Leydig cells and 10 μM concentration of ECH and RES has significantly reduced the expression of RAGE and NF-κB. The expression of NF-κB was significantly lower in RES and ECH than RAGE antagonist. So, the results confirmed that the ECH reduced the level of inflammation by decreasing the level of RAGE and NF-κB.

#### 3.1.6. Effect of ECH on the Testosterone Synthesis pathway in AGE-Stimulated LC-540 Leydig cells.

**Figure 6:**
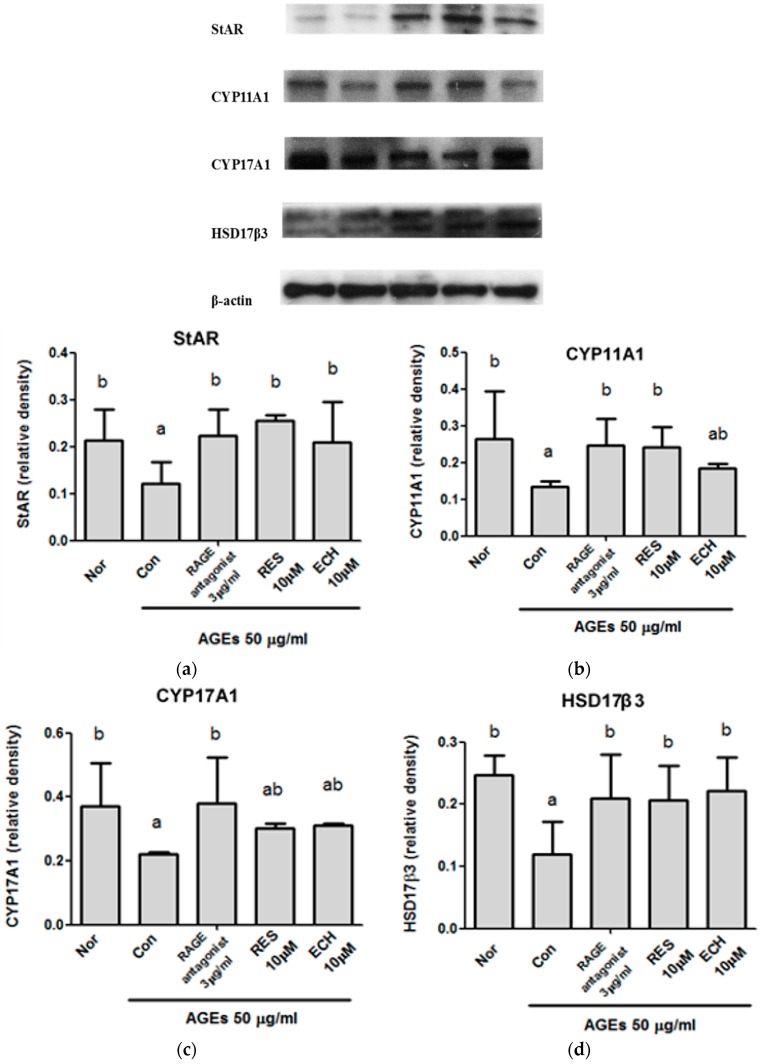
Effect of ECH on (**a**) steroidogenic acute regulatory protein (StAR), (**b**) cytochrome P450 11A1 (CYP11A1), (**c**) cytochrome P450 17A1 (CYP17A1), and (**d**) 17-beta hydroxysteroid dehydrogenase 3 (HSD17β3) protein expression levels in the testosterone synthesis pathway in AGE-stimulated LC-540 Leydig cells. The cell number was adjusted to 2 × 10^5^ cells/mL and treatment with RES and ECH (10 μM) was administered for 24 h. β-actin was taken as the internal control. The normal group (Nor) was treated with medium and the control group (Con) was treated with AGEs (50 μg/mL). Results were shown as mean ± SD (*n* = 3). Significant differences (*p* < 0.05) were represented by letters (a,b) analyzed by Duncan’s multiple range test.

The process of spermatogenesis and male infertility depends upon the presence of testosterone. As shown in Figure 6, the expressions of StAR (Figure 6a), CYP11A1 (Figure 6b), CYP17A1 (Figure 6c), and HSD17β3 (Figure 6d) proteins were significantly decreased in AGE-stimulated LC-540 Leydig cells (control group). The expressions of StAR, CYP11A1, CYP17A1, and HSD17β3 proteins were significantly increased when the RAGE antagonist, RES, and ECH were added. The increased expression of StAR and CYP11A1 proteins were seen in both the ECH- and RES-treated groups. The expression level of CYP17A1 was almost similar in RES and ECH groups. The expressions of the HSD17β3 protein in ECH-treated Leydig cells were much higher than those of the RES and RAGE antagonist-treated cells. Increased expression of StAR, CYP11A1, CYP17A1, and HSD17β3 proteins indicated the normal production of testosterone.

### 3.2. In Vivo Analysis

#### 3.2.1. Effects of CTE on Body Weight and Calorie Intake

**Figure 7:**
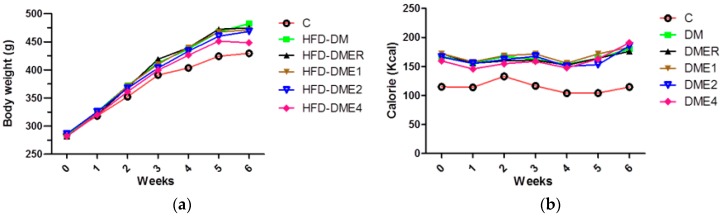
Effect of different concentrations of CTE on (**a**) body weight and (**b**) food intake of rats after 6 weeks of treatment. Data were shown as mean ± SD (*n* = 10 rats/group). The groups were as follows: control; DM (DM + 45% HFD), DMER (DM + rosiglitazone: 0.571 mg/kg BW + 45% HFD), DME1 (DM + CTE: 80 mg/kg BW + 45% HFD), DME2 (DM + CTE: 160 mg/kg BW + 45% HFD), DME4 (DM + CTE: 320 mg/kg BW + 45% HFD). Differences were considered significant at *p* < 0.05. HFD: high-fat diet; C: control; DME: diabetes mellitus + CTE; DMER: diabetes mellitus + rosiglitazone (RSG); DM: diabetes mellitus.

After 6 weeks of experimentation, the diabetic group (HFD-DM) showed higher body weight than the control group. HFD-DME4 group showed a lower body weight than the HFD-DM and HFD-DMER groups (Figure 7a). The calorie intake of the control group was significantly lower than in the other groups. There was no any significant difference between the calorie intakes of the other five groups (Figure 7b).

#### 3.2.2. Oral Glucose Tolerance Test (OGTT) to Determine the Successful Induction of Diabetes

**Figure 8:**
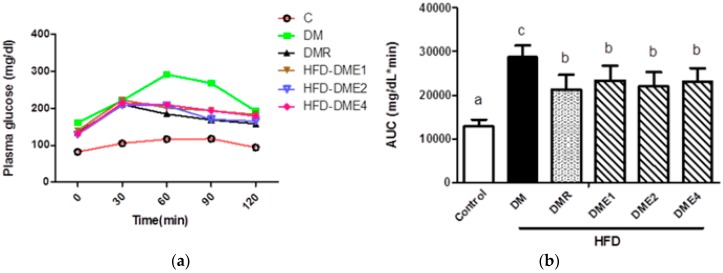
Effect of CTE on (**a**) oral glucose tolerance test (OGTT) (after oral glucose load of 2 g/kg) and (**b**) area under the curve (AUC) in rats fed different concentration after 6 weeks of treatment. Data were shown as the mean ± SD (*n* = 10 rats/group). The groups are follows: control; DM (DM + 45% HFD), DMER (DM + rosiglitazone: 0.571 mg/kg BW + 45% HFD), DME1 (DM + CTE: 80 mg/kg BW + 45% HFD), DME2 (DM + CTE: 160 mg/kg BW + 45% HFD), DME4 (DM + CTE: 320 mg/kg BW + 45% HFD). Differences were considered significant at *p* < 0.05 and denoted by letters (a,b). HFD: high-fat diet; C: control; DME: diabetes mellitus + CTE; DMR: diabetes mellitus + RSG.

Oral glucose tolerance test (OGTT) is used as a promising tool for detecting diabetic mellitus. Increased level of glucose in the blood indicating the diabetic condition. As shown in Figure 8a, the plasma glucose level was lower in CTE groups than in the DM group at 0, 30, 90, and 120 min. Further, the area under the curve (AUC) of plasma glucose concentration showed that in CTE and RSG groups, the blood glucose uptake rate was increased (Figure 8b).

#### 3.2.3. Total Plasma Glucose, Cholesterol, and Triglyceride Contents

The plasma fasting blood glucose level was higher in the DM group and lower in the DME2 group (except control) than others. There was no significant difference in the total cholesterol between the groups except for the DME4 group. In DME4 group, the cholesterol level was lower than the others. The level of triglycerides was higher in DM group and lower in DME4 group and the triglyceride content was decreased with increase in the concentration of CTE (Table 2). The results show that the level of plasma glucose, cholesterol, and triglycerides were higher in DM group and the level was significantly decreased on treatment with CTE.

#### 3.2.4. Plasma Insulin Levels, Plasma Leptin Level, and Homeostasis Model Assessment–Insulin Resistance (HOMA-IR) Values

The levels of plasma insulin, leptin, and HOMA-IR values are shown in Table 3. The DM group had higher plasma insulin and plasma leptin levels than the control group. The HOMA-IR index was also significantly higher in the DM group. The plasma insulin, leptin, and HOMA-IR values were decreased with increase in the concentration of CTE. Plasma leptin was significantly reduced in CTE groups but RSG drug group (DMR) did not show any significant difference from the DM group.

#### 3.2.5. Effect of CTE on Plasma LH and Testosterone Levels in Diabetic Rats

As shown in Table 4, the concentrations of testosterone in diabetic rats (DM) were significantly decreased, while the concentrations of testosterone were significantly increased at various doses of CTE. Further, the results showed a slight decrease in the level of LH in the DM group as compared to DMR, DME1, DME2, and DME4. The LH production was higher in DME4 group.

#### 3.2.6. Effect of CTE on the Sperm Parameters of Diabetic Rats

The experimental results showed that the DM group had a significant decrease in sperm number and motility than the control group whereas the sperm abnormality rate was significantly increased in the DM group. Interestingly, the sperm number, sperm motility, and the sperm abnormality rate of the DMR group improved but the sperm motility does not reach a significant level as compared to DME4. DME2 showed better sperm count than all of them and the motility rate was significantly increased in DME4 group. There was no any significant difference between the numbers of abnormal sperm between RSG- and CTE-treated groups (Table 5).

#### 3.2.7. Effect of CTE on the Morphology of Seminiferous Tubules in Diabetic Rats

**Figure 9:**
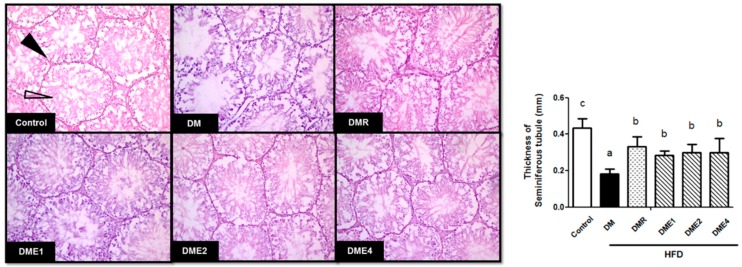
The testis sections of SD rats in each group were stained with hematoxylin and eosin after the experiment (magnifications = 200×. Differences were considered significant at *p* < 0.05 and denoted by letters (a–c). Leydig cells and Sertoli cells were indicated by black and white arrows, respectively.

Figure 9 shows the H&E staining of the testis section. The black arrow denotes the Leydig cell and the white arrow denotes the Sertoli cell. Both the Leydig cell and the Sertoli cell in the DM group showed significant atrophy and a cavity was seen in the lumen. The structure of Leydig cells and Sertoli were restored in CTE- and RSG-treated groups. The thickness of the seminiferous tubule was higher in the CTE and RSG groups than in the DM group.

#### 3.2.8. Effect of CTE on KiSS1, GPR54, SOCS-3, and SIRT1 mRNAs in the Hypothalamus of Diabetic Rats

**Figure 10:**
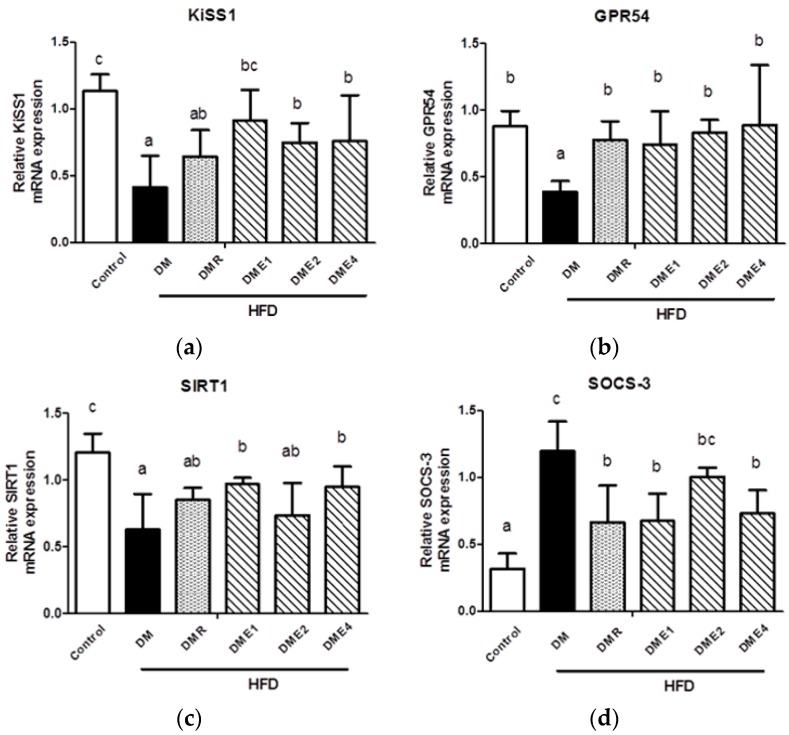
Expression of (**a**) KiSS1 mRNA (**b**) GPR54 mRNA (**c**) SIRT1 mRNA (**d**) SOCS-3 mRNA in the hypothalamus of rats after being fed with CTE for 6 weeks. Data were shown as the mean ± SD (*n* = 10 rats/group). Differences were considered significant at *p* < 0.05 and denoted by letters (a–c). HFD: high-fat diet; C: control; DME: diabetes × CTE; DMR: diabetes × RSG.

The expression of KiSS1 (Figure 10a), GPR54 (Figure 10b), SOCS-3 (Figure 10d), and SIRT1 (Figure 10c) were shown in Figure 10. The mRNA expression of KiSS1 and receptor GPR54 in diabetic rats was significantly lower than in the control group. The KiSS1 and GPR 54 mRNA expression level in DMR, DME1, DME2, and DME4 was significantly increased. In particular, the GPR54 mRNA expression was significantly increased in DME4 and was almost similar to the control group.

This experiment further explored the amount of SOCS-3 and SIRT1 mRNAs in the hypothalamus of rats. The expression of SOCS-3 mRNA in diabetic rats was increased significantly indicating that the leptin impedance was more serious. The DMR, DME1, DME2, and DME4 group showed significant improvements when compared to the diabetic group. The SIRT1 mRNA expression in the DM group was decreased significantly and was significantly increased in the DME1 and DME4 groups.

#### 3.2.9. Effect of CTE on Antioxidant Enzymes in Plasma and Testis of Diabetic Rats

Table 6 shows that the plasma SOD activity, GPx activity, and catalase activity of diabetic rats were decreased significantly and the activities were increased in CTE- and RSG-treated groups. In addition to this, DME4 showed significant improvements in GPx activity than others. Results showed that the SOD and catalase activities in the diabetic rats were significantly decreased after six weeks. The SOD and catalase activity of DMR group does not reach a significant level. The CTE-treated groups showed significant improvements in the SOD and catalase activity. The increased activities of catalase and SOD in CTE groups were also seen in testis (Table 7). The highest activity of SOD and catalase were observed in DME1 group and DME2 groups respectively.

#### 3.2.10. Effects of CTE on Oxidative Stress and Inflammation in the Plasma and Testis of Diabetic Rats

**Figure 11:**
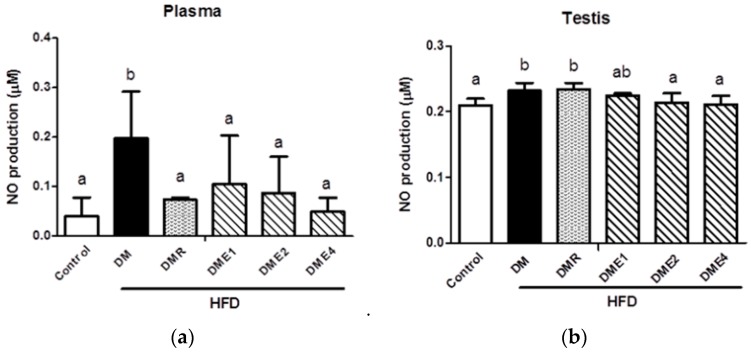
NO production in (**a**) plasma and (**b**) testis of diabetic rats fed with CTE for 6 weeks. Data were shown as the mean ± SD (*n* = 10 rats/group). Differences were considered significant at *p* < 0.05 and denoted by letters (a,b). HFD: high-fat diet; C: control; DME: diabetes mellitus + CTE; DMR: diabetes mellitus + RSG.

Nitric oxide (NO) production in plasma (Figure 11a) and testis (Figure 11b) were shown in Figure 11. The production of NO in DM group was significantly increased both in testis and plasma as compared to the control group. A gradual reduction of NO production was observed in DME1, DME2, and DME4 groups (in plasma). The DMR group also showed a significant reduction in NO production. In the case of the testis, there was a slight reduction of NO production was observed in CTE groups, whereas the DMR group does not significantly reduce NO production.

**Figure 12:**
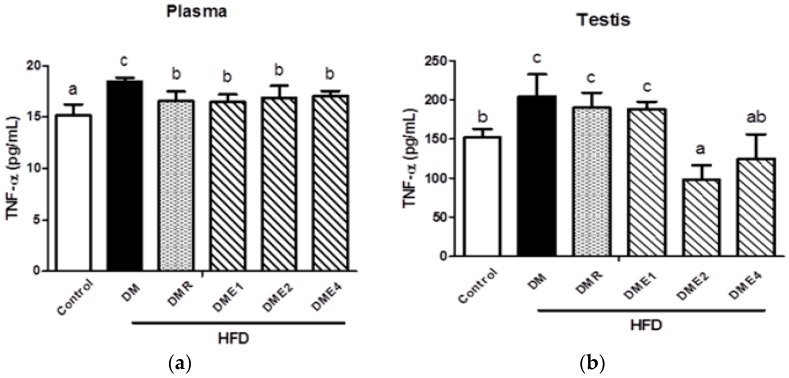
The levels of TNF-α in (**a**) plasma and (**b**) testis of diabetes rats after being fed with CTE for 6 weeks. Data were shown as the mean ± SD (*n* = 10 rats/group). Differences were considered significant at *p* < 0.05 and denoted by letters (a–c). HFD: high-fat diet; C: control; DME: diabetes mellitus + CTE; DMR: diabetes mellitus + RSG.

**Figure 13:**
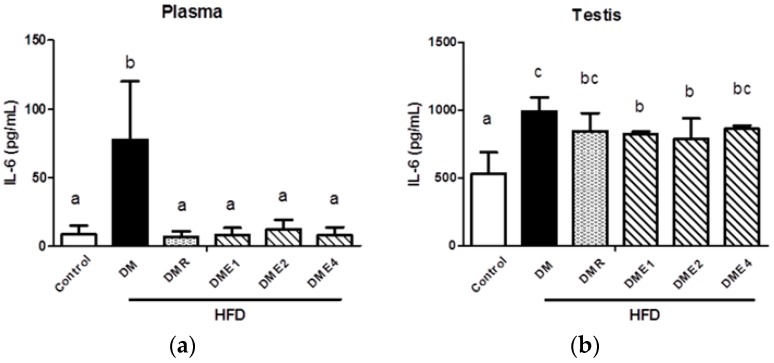
The levels of interleukin 6 (IL-6) in (**a**) plasma and (**b**) testis of diabetes rats after being fed with CTE for 6 weeks. Data were shown as the mean ± SD (*n* = 10 rats/group). Differences were considered significant at *p* < 0.05 and denoted by letters (a–c). HFD: high-fat diet; C: control; DME: diabetes mellitus + CTE; DMR: diabetes mellitus + RSG.

As shown in Figure 12 and Figure 13, the level of TNF-α and IL-6 were significantly increased in diabetic rats (both in plasma and testis), indicated that the inflammation is more serious. In plasma, the level of TNF-α is similar in the CTE- and RSG-treated groups (Figure 12a). The CTE groups significantly (especially DME2) reduced the level of TNF-α in the testis (Figure 12b). The level of IL-6 was significantly reduced in the plasma of CTE and RSG groups (Figure 13a). In the testis, there was a trend to a reduction in the level of IL-6, but this did not reach a significant level (Figure 13b).

#### 3.2.11. Effects of CTE on Oxidative Stress and Inflammation in Spermatozoa of Diabetic Rats Induced by High-Fat Diet

**Figure 14:**
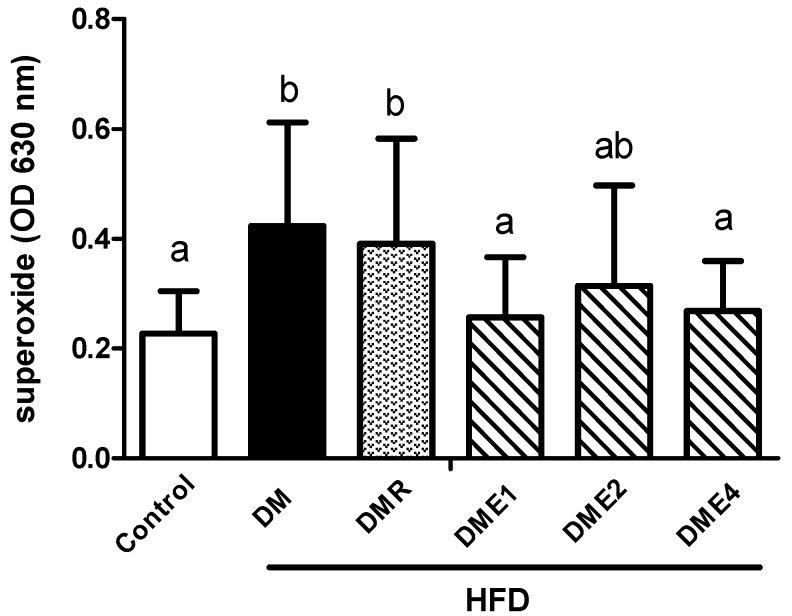
The level of superoxide production in the sperm of diabetic rat after feeding with CTE for 6 weeks (NBT assay). Data were shown as the mean ± SD (*n* = 10 rats/group). Differences were considered significant at *p* < 0.05 and explained by letters (a,b). HFD: high-fat diet; C: control; DME: diabetes mellitus + CTE; DMR: diabetes mellitus + RSG.

Figure 14 shows the superoxide anion content in rat sperm. The results showed that the superoxide anion production in the sperm of diabetic rats increased significantly and there was no significant improvement was observed in the DMR group. DME1 and DME4 groups showed significantly reduced production of superoxide anion.

#### 3.2.12. Effects of CTE on Lipid Peroxidation in Spermatozoa of Diabetic Rats Induced by High-Fat Diet

Studies have been shown that the lipid peroxidation was increased in both type 1 or type 2 diabetes patients [19]. The malondialdehyde (MDA) level in plasma, sperm, and testis was shown in Table 8. MDA level in plasma, testis, and sperm of DM group was significantly higher and the treatment with CTE and RSG reduced the production of MDA. A significant reduction was observed in DME4 and DMEI groups in plasma. This study found that the diabetic rats not only increased the degree of lipid peroxidation in plasma but also increased in testis and sperm. Significant improvement was observed at various doses of CTE.

## 4. Discussion

Diabetes is a chronic disease associated with a high level of sugar in the blood. The imbalance between antioxidant and ROS level will lead to the condition called oxidative stress. Superoxide, hydroxyl radical, hydrogen peroxide, nitric oxide, and singlet oxygen are some of the examples of ROS that will contribute diabetes condition via oxidative stress [20]. *Cistanche tubulosa* is a desert plant contains active components such as polysaccharides, oligosaccharides, phenylethanoid glycosides (echinacoside, verbascoside), palmitic acid, linoleic acid, iridoids, alditols, and lignans. This plant is capable of producing anti-inflammatory, neuroprotective, antibacterial, antiviral, anti-oxidative, anti-tumor, and immunomodulatory effects [21]. Literature studies show that the phenylethanoid glycosides from *Cistanche tubulosa* are the major reason for the antioxidant activity [22]. Resveratrol (RES) and rosiglitazone (RSG) were taken as the positive control for in vitro and in vivo studies respectively. RSG is a powerful insulin sensitizer and has an affinity towards the isoform of peroxisome proliferator activated receptor (PPARc). It controls hyperglycemia in diabetic patients [23]. RES have natural antioxidant activity, acts as a vasodilator, regulating lipoprotein metabolism, inhibiting platelet aggregation, and preventing cancer [24,25]. Our investigation indicated that the ECH show better radical scavenging activity than CTE and RES (Figure 1). It is also understood that the ECH does not cause any significant toxicity to LC-540 and TM3 Leydig cells (Figure 2).

AGEs were used to induce stress condition in Leydig cells. The production of AGEs, inflammation, oxidative stress, and diabetes are interconnected. The hyperglycemic condition of diabetes promotes cell injury through the production of AGEs and oxidative stress. AGEs induce β cell toxicity, which further promotes immune cell recruitment and cell death. An elevated level of AGEs promotes the expression of two type of receptors called RAGE and AGER1 [26]. The dripping and subsequent transfer of an electron from the mitochondrial respiratory chain to molecular oxygen during oxidative stress results in the formation of superoxide anion. Our results show that the production of superoxide anion induced by AGEs was increased in the control group and further improvements were observed with both ECH and RES treatments. According to our study, it was understood that the superoxide (Figure 3) and H_2_O_2_ (Figure 4) production induced by AGEs was decreased in the presence of ECH and RES.

NF-κB is known as the important mediator of inflammation associated with diabetes [27]. The expression of NF-κB lead to β cell dysfunction and cell death. The activation of NF-κB by oxidative stress stimulates the pro-inflammatory response, upregulation of endothelin, and apoptosis [28]. The expression of RAGE and NF-κB were increased in AGE-stimulated groups and the subsequent reduction was observed in ECH- and RES-treated cells (Figure 5).

Testosterone is an anabolic steroid and primary male sex hormone synthesized from cholesterol. The process starts with the oxidative cleavage of cholesterol’s side chain by cholesterol side chain cleavage gene (*CYP11A*). This gene is localized in the mitochondrial membrane and convert the cholesterol into pregnenolone. Next, the CYP17A1 gene from the endoplasmic reticulum removes two extra carbon atoms and produce multiple C19 steroids. In addition to this, the pregnenolone is oxidized to form androstenedione/progesterone by hydroxysteroid dehydrogenase (3-β-HSD). Finally, the testosterone is produced by the reduction of keto group in the 17th carbon position of androstenedione by 17-beta-hydroxysteroid dehydrogenase (17-β-HSD). Leydig cells are involved in the major production of testosterone. The transfer of cholesterol into the inner mitochondrial membrane requiring the action of steroidogenic acute regulatory protein (StAR). Present studies show that the expression of StAR, CYP11A1, CYP17A1, and HSD17β3 were decreased in AGE-treated cells (control) and there was a tremendous increase were shown in ECH- and RES-treated cells (Figure 6). Thus, the results show that the production of testosterone was increased in the ECH- and RES-treated groups.

During a diabetic condition, the transport of glucose to organs is limited and as a result, the glucose levels increase [29]. Hence, the hyperglycemic condition is one of the important markers for diabetic detection. Our studies show that the plasma glucose level was increased in diabetic groups and the AUC of the DM group were significantly higher than the other groups (Figure 8). Along with plasma glucose, the total cholesterol and triglyceride content was significantly increased in the DM group (Table 2). Atherosclerosis is facilitated by an increased level of cholesterol and triglycerides [30]. However, the level of cholesterol and triglycerides were decreased in CTE- and RSG-treated groups. The triglyceride content of DME4 group reached almost similar value with the control group.

Insulin is a hormone produced by the β cells of the pancreas and functionalized to control blood sugar level in the body while leptin is a hormone produced by the adipocytes and are capable of regulating the food intake and energy utilization. Studies indicated that the leptin is involving in the pathophysiology of obesity and there is a positive interaction between leptin and insulin [31]. Our studies show that the level of plasma inulin and leptin were higher in DM group and the insulin level was decreased with increase in the concentration of CTE. The RSG group does not show any significant difference from the DM group. The insulin resistance and β cell function were assessed by HOMA-IR method. The HOMA-IR value increased in the DM group and was significantly different from other groups (Table 3).

DM affect the reproductive function via the hormonal alternation in the HPG axis and studies revealed that the insulin expression in the testis is also affected by diabetes. It is characterized by Sertoli cell vacuolization, increased DNA fragmentation, impaired spermatogenesis, and increased germ cell depletion. Oxidative stress also contributes to abnormalities in reproductive function [32]. The process of formation of sperm in the male reproductive organ (testes) is called spermatogenesis. The testis is composed of tightly coiled tubules called seminiferous tubules. Sertoli cells are seen in the walls of the seminiferous tubule and provide nourishment to the immature sperm. The investigation on sperm parameters indicated that the number of sperm and motility decreased and the abnormalities increased in DM group (Table 5). An opposite effect was observed in the CTE- and RSG-treated groups. Both the Leydig cell and the Sertoli cells in the DM group showed significant atrophy and the cavity was seen in the lumen. The thickness of the seminiferous tubule also decreased in the DM group. An improved result was observed in CTE- and RSG-treated groups (Figure 9). In addition to this, the level of LH and testosterone decreased in the DM group and the levels increased in CTE-treated groups (Table 4). In men, low serum testosterone and lower LH pulse frequency were often associated with obesity and diabetes mellitus type 2 [33].

Kisspeptins encoded by the KiSS1 gene are known as the potent stimulator of the HPG axis and any mutation in the kisspeptin gene will lead to low levels of sex steroids and gonadotropin. Studies show that in STZ-induced diabetic rats, the Kiss1 mRNA levels were decreased [33]. The initiation and maintenance of mammalian infertility are connected with G-protein coupled receptor 54 (GPR54). The mutation in GPR 54 is characterized by the absence of sexual maturation and low levels of gonadotropic hormones (LH and FSH). Pro-inflammatory cytokines such as IL-6 and TNF-α up-regulate the expression of the suppressor of cytokine signaling 3 (SOCS3) implicated in inflammation-mediated insulin resistance in the liver and adipocytes [34]. SIRT 1 is a gene associated with the regulation of several aging diseases. This gene is prominently expressed in the β cells of the pancreas and regulate insulin secretion and preventing apoptosis. Current studies indicated that the expression of KiSS1, GPR54, and SIRT1 decreased in the DM group, but the expression increased in CTE-treated groups (Figure 10). Increase in the SOCS-3 expressions in the DM group indicating the inflammatory condition. The first molecular link identified between obesity and inflammation was TNF-α. Hence, an increased level of TNF-α is an indicator of inflammation. The decreased level of pro-inflammatory cytokines such as TNF-α and IL-6 were seen in CTE-treated groups (Figure 12 and Figure 13).

The imbalance between ROS and antioxidants leads to the diabetic condition. Superoxide dismutase (SOD), catalase (CAT), and glutathione peroxidase (GPX) are known as the primary antioxidants responsible for maintaining the optimum ROS level [35]. From the results, it was found that the activity of antioxidants was significantly lower in the DM group. CT-treated groups shown improvements in the production of antioxidants. The RSG-treated group did not show any significant improvements in antioxidant activity (Table 6 and Table 7).

Nitric oxide (NO) is known as an important ROS that contributes to inflammation. Our studies point out that the level of NO was decreased in CTE-treated groups (Figure 11). The results showed that the superoxide anion content in the sperm of diabetic rats increased significantly and there was no any significant improvement after the administration of RSG. The production of superoxide was reduced in CTE groups.

The determination of MDA is very useful for evaluating lipid peroxidation. Lipid peroxidation is the process of oxidation in lipids and finally results in cell damage. MDA is produced as the result of lipid peroxidation of polyunsaturated fatty acids. Studies show that MDA level is correlated with age and fasting blood glucose level [36]. The current study indicated that the CTE improves lipid peroxidation in plasma, testis, and sperm (Table 8).

## 5. Conclusions

The oxidative stress during diabetic condition disturbs the male reproductive system by sperm impairment and gonadal dysfunction. *Cistanche tubulosa* is a desert plant widely accepted in Chinese medicine due to its pharmacological effects. Echinacoside (ECH) is the main constituent of CTE responsible for the antioxidant and anti-inflammatory activities. Our in vitro results indicated that the ECH restored the testosterone synthesis pathway and lowering the level of NF-κB and RAGE protein expression. ECH effectively inhibited the production of superoxide anion and H_2_O_2_ in Leydig cells. The in vivo studies revealed that the ECH reduced the levels of cholesterol, triglycerides, TNF-α, and IL-6. In addition to this, the mRNA expressions in the hypothalamus of the diabetic rats were significantly improved. It is also of note that the ECH reduced lipid peroxidation and improved insulin resistance in diabetic male rats. The antioxidant activities increased both in plasma and testis. Therefore, our studies suggested that the ECH provided effective protection against the reproductive dysfunction in STZ-induced diabetic male rats.

## Figures and Tables

**Table 1:** List of primers used in the experiment.

Gene Bank Accession Number	Expected Size (bp)
*Kiss1* sense (5′-ATGATCTCGCTGGCTTCTTG-3′) *Kiss1* antisense (5′-AGGCTTGCTCTCTGCATACC-3′)	174
*Gpr54* sense (5′-CTGGGAGACTTCATGTGCAA-3′) *Gpr54* antisense (5′-GAACCCACCCAGATGCTAAG-3′)	182
*Socs-3* sense (5′-GTC GGGGACCAAGAACCTAC-3′) *Socs-3* antisense (5′-GGCTGCTCCTGAACCTCAAA-3′)	192
*Sirt1* sense (5′-ATTTATGCTCGCCTTGCTGT-3′) *Sirt1* antisense (5′-GATCCTTTGGATTCCTGCAA-3′)	243
Internal Control Gene	
*Rp-s11* sense (5′-GGCGGACATTCAGACGGAG-3′) *Rp-s11* antisense (5′-CGTCACAACACCAGACAGGA -3′)	232

**Table 2:** Plasma fasting blood glucose, total cholesterol, and triglyceride in diabetic rats after being fed with different concentrations of CTE for 6 weeks.

mg/dL		HFD				
	C	DM	DMR	DME1	DME2	DME4
Fasting glucose	82.2 ± 13.68 ^a^	160.82 ± 19.71 ^c^	134.37 ± 17.49 ^b^	141.68 ± 16.41 ^b^	129.23 ± 18.74 ^b^	131.04 ± 15.68 ^b^
Total cholesterol	101.30 ± 27.92 ^b^	104.26 ± 20.92 ^b^	106.90 ± 19.26 ^b^	101.85 ± 19.64 ^b^	104.51 ± 19.78 ^b^	81.08 ± 11.58 ^a^
Triglyceride	60.82 ± 15.88 ^a^	87.07 ± 22.42 ^b^	62.77 ± 16.05 ^a^	78.46 ± 29.85 ^ab^	73.44 ± 17.88 ^ab^	56.29 ± 14.74 ^a^

Data were shown as the mean ± SD (*n* = 10 rats/group). Differences were considered significant at *p* < 0.05. HFD: high-fat diet; C: control; DME: diabetes + CTE; DMR: diabetes + RSG. Differences were considered significant at *p* < 0.05 and denoted by letters (a,b).

**Table 3:** Plasma insulin levels, leptin level and Homeostasis model assessment equation (HOMA-IR) in diabetic rats after fed with different concentration of CTE for 6 weeks.

ng/mL		HFD				
	C	DM	DMR	DME1	DME2	DME4
Insulin	3.33 ± 1.83 ^a^	5.41 ± 2.28 ^b^	3.57 ± 2.15 ^a^	3.17 ± 1.74 ^a^	2.34 ± 2.03 ^a^	2.28 ± 1.43 ^a^
Leptin	2.97 ± 1.51 ^a^	7.36 ± 1.76 ^b^	6.38 ± 2.05 ^b^	6.26 ± 2.68 ^b^	3.66 ± 3.05 ^a^	3.57 ± 1.71 ^a^
HOMA-IR (µg.mmol/L^2^)	1.38 ± 0.70 ^a^	4.43 ± 1.87 ^b^	2.37 ± 1.21 ^a^	2.45 ± 1.79 ^a^	1.62 ± 1.57 ^a^	1.59 ± 1.04 ^a^

Data were shown as the mean ± SD (*n* = 10 rats/group). Differences were considered significant at *p* < 0.05 and denoted by letters (a,b). HOMA- IR: Homeostasis Model Assessment–Insulin Resistance; HFD: high-fat diet; C: control; DME: diabetes mellitus + CTE; DMR: diabetes mellitus + RSG.

**Table 4:** Effect of CTE on plasma hormone level after 6 weeks of treatment in diabetic rats. Data were shown as the mean ± SD (*n* = 10 rats/group).

		HFD				
	C	DM	DMR	DME1	DME2	DME4
LH (IU/L)	6.20 ± 0.09 ^b^	6.09 ± 0.01 ^a^	6.17 ± 0.06 ^b^	6.18 ± 0.02 ^b^	6.16 ± 0.02 ^b^	6.22 ± 0.09 ^b^
Testosterone (ng/dL)	3.84 ± 1.04 ^d^	1.30 ± 0.33 ^a^	1.95 ± 0.69 ^b^	3.51 ± 0.43 ^c^	2.43 ± 0.24 ^bc^	2.64 ± 0.68 ^bc^

Differences were considered significant at *p* < 0.05. LH: luteinizing hormone; HFD: high-fat diet; C: control; DME: diabetes mellitus + CTE; DMR: diabetes mellitus + RSG. Differences were considered significant at *p* < 0.05 and denoted by letters (a–c).

**Table 5:** Effect of CTE on sperm parameters in diabetic rats after 6 weeks. Data were shown as the mean ± SD (*n* = 10 rats/group).

Sperm		HFD				
	C	DM	DMR	DME1	DME2	DME4
Total count (*10^5^)	21.50 ± 4.34 ^b^	16.60 ± 1.50 ^a^	31.30 ± 4.96 ^c^	32.50 ± 8.02 ^c^	38.80 ± 7.85 ^d^	23.10 ± 7.69 ^b^
Motility (% total)	21.70 ± 12.43 ^b^	11.74 ± 12.03 ^a^	15.83 ± 7.09 ^ab^	20.54 ± 7.83 ^b^	14.22 ± 1.87 ^b^	49.60 ± 3.88 ^c^
Abnormal morphology (% total)	5.00 ± 4.28 ^a^	16.57 ± 18.59 ^b^	3.28 ± 3.90 ^a^	4.37 ± 3.21 ^a^	3.09 ± 3.91 ^a^	5.76 ± 7.23 ^a^

Differences were considered significant at *p* < 0.05 and denoted by letters (a–c). HFD: high-fat diet; C: control; DME: diabetes mellitus + CTE; DMR: diabetes mellitus + RSG.

**Table 6:** Effect of CTE on plasma anti-oxidative enzyme activity after 6 weeks of treatment in diabetic rats.

	(Units/mg Protein)					
		HFD				
	C	DM	DMR	DME1	DME2	DME4
SOD	0.60 ± 0.13 ^b^	0.40 ± 0.05 ^a^	0.65 ± 0.15 ^b^	0.64 ± 0.24 ^b^	0.63 ± 0.25 ^b^	0.55 ± 0.03 ^ab^
Catalase	81.58 ± 32.37 ^b^	44.07 ± 6.79 ^a^	60.05 ± 9.92 ^ab^	68.24 ± 27.80 ^b^	70.53 ± 33.79 ^b^	58.29 ± 8.50 ^ab^
GPx	1974.36 ± 609.87 ^c^	606.41 ± 498.49 ^a^	811.08 ± 359.72 ^ab^	918.66 ± 228.38 ^ab^	948.06 ± 501.99 ^ab^	1065.95 ± 168.89 ^b^

Data were shown as the mean ± SD (*n* = 10 rats/group). Differences were considered significant at *p* < 0.05 and denoted by letters (a–c). SOD: superoxide dismutase; GPx: glutathione peroxidase; HFD: high-fat diet; C: control; DME: diabetes mellitus + CTE; DMR: diabetes mellitus + RSG.

**Table 7:** Effect of CTE on testis anti-oxidative enzyme activity after 6 weeks of treatment in diabetic rats.

	(Units/mg Protein)					
		HFD				
	C	DM	DMR	DME1	DME2	DME4
SOD	17.29 ± 5.13 ^b^	13.74 ± 2.01 ^a^	13.55 ± 3.08 ^a^	18.26 ± 4.27 ^b^	16.30 ± 1.18 ^ab^	16.16 ± 2.12 ^ab^
Catalase	416.69 ± 80.41 ^b^	213.39 ± 126.59 ^a^	205.06 ± 114.58 ^a^	393.95 ± 202.83 ^ab^	426.55 ± 76.79 ^b^	421.61 ±144.60 ^b^

Data were shown as the mean ± SD (*n* = 10 rats/group). Differences were considered significant at *p* < 0.05 and denoted by letters (a,b). SOD: superoxide dismutase; HFD: high-fat diet; C: control; DME: diabetes mellitus + CTE; DMR: diabetes mellitus + RSG.

**Table 8:** The effect of CTE on lipid peroxidation of plasma, testis, and sperm in diabetic rats after 6 weeks.

nmol/mL		HFD				
	C	DM	DMR	DME1	DME2	DME4
Plasma MDA	10.15 ± 1.20 ^a^	16.87 ± 2.42 ^c^	13.38 ± 2.12 ^b^	9.90 ± 2.09 ^a^	10.66 ± 1.94 ^a^	9.95 ± 1.55 ^a^
Testis MDA	10.16 ± 0.79 ^a^	13.31 ± 0.71 ^b^	11.64 ± 2.70 ^a^	11.75 ± 0.50 ^a^	11.54 ± 1.71 ^a^	11.30 ± 2.00 ^a^
Sperm MDA	1.39 ± 0.73 ^a^	2.12 ± 0.28 ^b^	1.75 ± 0.41 ^ab^	1.39 ± 0.78 ^a^	1.47 ± 0.50 ^a^	1.42 ± 0.82 ^a^

Data were shown as the mean ± SD (*n* = 10 rats/group). Differences were considered significant at *p* < 0.05 and denoted by letters (a–c). HFD: high-fat diet; C: control; DME: diabetes mellitus + CTE; DMR: diabetes mellitus + RSG.
